# Paradoxical exacerbation of myoclonic-astatic seizures by levetiracetam in myoclonic astatic epilepsy

**DOI:** 10.1186/s12887-015-0330-y

**Published:** 2015-02-12

**Authors:** Yamane Makke, Ghassan Hmaimess, Wassim Nasreddine, Ahmad Fawaz, Ahmad Beydoun

**Affiliations:** Department of Neurology, American University of Beirut Medical Center, Beirut, Lebanon; Department of Pediatric, St George Hospital Medical University Center, University of Balamand, Beirut, Lebanon

**Keywords:** Epilepsy, Electroclinical syndrome, Doose syndrome, Antiepileptic, Levetiracetam, Myoclonus

## Abstract

**Background:**

Levetiracetam is a broad spectrum antiepileptic drug (AED) with proven efficacy when used as adjunctive therapy against myoclonic seizures. We report two patients suffering from epilepsy with myoclonic-astatic epilepsy (MAE) who experienced a paradoxical worsening of seizures after initiation of treatment with LEV, a finding not previously described.

**Case presentation:**

Patients included were enrolled in an ongoing large prospective study evaluating children and adults with new onset epilepsy in Lebanon conducted at the American University of Beirut Medical Center in association with the Lebanese Chapter of the International League against Epilepsy. Based on an extensive evaluation, these patients were stratified into idiopathic partial, idiopathic generalized, symptomatic partial or symptomatic generalized epilepsies. Whenever possible the electroclinical syndrome was identified according to the ILAE classification of epilepsy syndromes. Patients were subsequently followed up on regular intervals and were assessed for adverse events, and seizure recurrence.

MAE was diagnosed in five (1.6%) out of 307 consecutive children enrolled in this study. LEV was used as adjunctive therapy in four of those children with two experiencing a substantial and dose related worsening in the frequency of their myoclonic and atonic seizures.

**Conclusion:**

LEV should be used with caution in children with MAE and an exacerbation of seizure frequency temporally related to the introduction of LEV should alert the clinician to the possibility of a paradoxical seizure exacerbation.

## Background

Levetiracetam (LEV), one of the new generation antiepileptic drugs (AEDs), is considered to be a broad spectrum anticonvulsant with favorable safety and pharmacokinetic profiles [1]. It is currently licensed in the USA as adjunctive therapy for the treatment of partial onset seizures in patients one month of age and older, myoclonic seizures in patients 12 years and older diagnosed with juvenile myoclonic epilepsy [2] and primarily generalized tonic-clonic seizures in patients 6 years and older diagnosed with an idiopathic generalized epilepsy [3].

MAE, first described as an independent epilepsy syndrome by Doose [4] is included in the international classification of epilepsy syndromes as one of the cryptogenic/symptomatic generalized epilepsies [5]. It was recently renamed as epilepsy with myoclonic-atonic seizures and classified as one of the childhood onset electroclinical syndromes [6]. It is a generalized epilepsy, typically affecting developmentally normal children with a mean age at onset of 2–4 years and is clinically characterized by the occurrence of myoclonic, atonic, myoclonic-atonic and absence seizures which are usually preceded by febrile or afebrile generalized tonic-clonic seizures. The EEG is characterized by a normal background with bursts of generalized 2–4 Hz spike or polyspike wave discharges.

LEV efficacy against myoclonic seizures has led to its off label use in a variety of conditions associated with myoclonus including post-anoxic myoclonus [7] and childhood epilepsies with myoclonic seizures [8-10]. We present two children diagnosed with epilepsy with myoclonic-astatic epilepsy (MAE) who experienced a substantial exacerbation of their myoclonic and atonic seizures following initiation of treatment with LEV and improvement following discontinuation of this AED. This paradoxical reaction to LEV in children with MAE was not previously reported.

## Case presentation

Patients identified in this report were enrolled in an ongoing prospective study on children and adults with newly diagnosed epilepsy. This is a centralized study conducted at the American University of Beirut Medical Center (AUBMC) in association with the Lebanese Chapter of the International League against Epilepsy. One of the principal aims of this study is to determine the frequency of epilepsy syndromes in Lebanon stratified according to age groups in patients with newly diagnosed epilepsy. Adult and pediatric neurologists from across Lebanon are referring patients with suspected newly diagnosed seizure/epilepsy to the AUBMC where a full history including a detailed description of the seizures is taken by an epileptologist and physical/neurological examinations are performed. The work-up on each patient includes a 3-hour sleep deprived video-EEG recording interpreted by two experienced electroencephalographers along with an epilepsy protocol brain MRI interpreted by a neuroradiologist blinded to the patient’s history. The results of those tests are then communicated to the referring physician who decides on whether to initiate treatment (if treatment not already initiated) and on the choice of AED. A baseline bone densitometry is performed on patients started on AED treatment. Patients are then followed up by monthly phone calls inquiring about medications adverse events, AED levels and seizure recurrence with follow up visits at the AUBMC every 3–6 months and repeat video-EEG recording and filling of quality of life questionnaires. This study is approved by the Institutional review Board of the AUBMC and all patients enrolled in the study signed an informed consent form (parental signature for children and adolescents).

Based on the results of the history, seizure semiology, physical examination, video/EEG and MRI findings and follow up (when needed), we stratified patients into idiopathic partial, idiopathic generalized, symptomatic partial or symptomatic generalized epilepsies and identified the electroclinical syndrome, whenever possible, based on the ILAE classification of epilepsy syndromes [5].

Out of first 307 children and adolescents (less than 18 years of age) with two or more unprovoked seizures enrolled in the study, five children (1.6%) were diagnosed with MAE. There were four boys and one girl, with a mean age of 3.3 years, and a range from 1.9 to 5.4 years. All patients were initially treated with valproate but failed to achieve seizure remission. Of the four children subsequently treated with add-on LEV, two had no change in seizure frequency while two others, which are the basis of this report, had a substantial exacerbation in the frequency of their myoclonic and atonic seizures following the introduction of this drug.

### Case 1

AA is a 3 years 3 months old girl, with a history of new onset unprovoked nocturnal and diurnal GTCs with no focal onset for which treatment with valproate was initiated. At her initial evaluation at AUBMC, she had a normal psychomotor development, normal physical and neurological examinations, and a history of epilepsy in a maternal uncle as her only risk factor for epilepsy. A three-hour sleep deprived EEG revealed a normal awake background rhythm, and fragments of generalized spike and wave discharges. Brain MRI was normal except for a non-specific faint high FLAIR signal in the peritrigonal white matter. Two months later, the patient started to develop myoclonic jerks of the upper extremities associated with hiccup noise followed by head drops and falls five times daily (the parents described the child as limp when falling) resulting in multiple head and bodily injuries. A repeat three-hour video/EEG recording revealed the presence of frequent, 2.5-3.5 Hz generalized, frontally predominant spike and wave discharges. Treatment with LEV was initiated at 250 mg BID (31 mg/kg/day). Three days later, the parents noted that the frequency of the myoclonic jerks followed by head drop and falls increased to 10 times daily and then to 15 times daily when the dose of LEV was further increased to 375 mg BID (47 mg/kg/day). The dose of LEV was reduced to 250 mg BID with a rapid reduction of myoclonic-atonic seizures back to 10 times daily. LEV was tapered and discontinued and treatment with ethosuximide was initiated with complete resolution of her seizures and complete normalization of her EEG.

### Case 2

WS is a 22 months old boy, born after an uneventful pregnancy and with normal psychomotor development, who presented with a history of 6 unprovoked afebrile generalized tonic-clonic seizures over a period of 10 days. The parents also reported noticing multiple daily staring episodes, each lasting for approximately 10 seconds and associated with unresponsiveness and blinking. At the time of his presentation to the AUBMC, treatment with valproate was already initiated by the referring pediatric neurologist. His physical and neurological examinations were normal. There were no risk factors for epilepsy. A three-hour sleep deprived video/EEG recording revealed bursts of frontally predominant, 2–3 Hz generalized spike and wave discharges lasting typically between 2 and 5 seconds. Some discharges lasted up to 17 seconds and were clinically associated with absences. Brain MRI was normal. Due to the persistence of absence seizures, add-on treatment with LEV was initiated at 125 mg BID (21 mg/kg/day) by the referring physician. Two days later, the child started to experience brief myoclonic jerks, recurring in clusters and resulting in brief extension of the neck. As the dose of LEV was uptitrated to 500 mg/day (42 mg/kg/day), the parents started noticing frequent head drops (averaging 3–4 episodes/day) without falls sometimes preceded by myoclonic jerks of the upper extremities, but resolution of the staring episodes. A repeat EEG again showed short bursts of frontally predominant generalized irregular 2–3 Hz spike and wave activity and a myoclonic-astatic seizure was recorded. The dose of LEV was further increased to 750 mg/day (63 mg/kg/day) with a substantial increase in the frequency of the head drops, with an average of 7 such seizures witnessed by the parents on a daily basis. Reduction in the dose of LEV to 500 mg/day without change in concomitant medication resulted in a reduction in the frequency of the head drops to 3 events per day within 24 hours of the reduction in the LEV dose. Subsequently, treatment with ethosuximide was initiated and LEV was tapered and discontinued. This treatment regimen resulted in total cessation of seizures and total normalization of the EEG.

## Conclusions

In both children, the introduction of LEV resulted in an exacerbation of the myoclonic and atonic seizures. Drug induced exacerbation of seizures are difficult to detect clinically because of the natural history of epilepsy, with periods of seizure exacerbations unrelated to a particular drug regimen. A causal relationship between an AED and seizure exacerbation is best established when it can be demonstrated that the introduction of that AED resulted in increased seizure frequency, its discontinuation resulted in reduction in seizure frequency, and that a rechallenge with the AED resulted again in a worsening of seizure frequency. In our patients, although reintroduction of LEV was not done because of ethical reasons, a causal relationship is strongly suspected not only because of the close temporal relationship between the introduction of LEV and worsening seizure frequency but also because of the dose-related exacerbation in seizure frequency.

In our first case, the child developed myoclonic-atonic seizures a couple of month after the onset of afebrile generalized tonic-clonic seizures, a typical sequence for patients with MAE. A few days after the introduction of LEV, the daily frequency of myoclonic-atonic seizures that resulted in falls increased and got proportionately worse as the dose of LEV was further increased and improved with dose reduction.

In our second case, the appearance of myoclonic-atonic seizures shortly after the introduction of LEV could very well have been a chance association and a reflection of the natural course of the disease. However, there was again a very clear exacerbation in the frequency of the mycolonic-atonic seizures that correlated with progressive increase in the dose of LEV and a subsequent amelioration with dose reduction.

In both children, withdrawal of LEV and introduction of ethosuximide resulted in total seizure remission and normalization of the EEG. MAE is notoriously a difficult condition to treat. Although no clinical trial evaluating the safety and efficacy of AEDs in MAE has been conducted, ethosuximide [11] and valproate [12,13] are usually advocated as first-line therapy, although the percentage of children achieving a remission with valproate monotherapy is quite low [14]. Other drugs that have been anecdotally tried include lamotrigine, zonisamide, LEV, and rufinamide [11,15]. The ketogenic diet is particularly effective for children with MAE, and although typically used in children refractory to drug treatment, some have advocated for its use earlier in the clinical course [16-18].

The efficacy of LEV in MAE was only reported in 9 children, with unimpressive results since only one was reported to achieve seizure freedom with a follow-up of only 3 months [8,11,14]. Our data is consistent with those results since two of the 4 children treated with LEV had no significant change in seizure frequency while the other two experienced a significant worsening of their myoclonic and atonic seizures, a paradoxical reaction not previously described for LEV in MAE. There is only one reported patient with MAE incompletely controlled on valproate monotherapy who reportedly developed myoclonic status epilepticus after replacing valproate with LEV [19]. This worsening could have been due to the discontinuation of valproate, introduction of LEV, natural history of the illness or any combination of those factors.

A number of AEDs, including carbamazepine, phenytoin and vigabatrin should be avoided in MAE because they frequently result in a significant exacerbation of seizures [11]. In addition, although the combination of valproate and lamotrigine is favored by some as a synergistic combination in this condition [20,21], lamotrigine should be used cautiously in MAE especially in children with frequent myoclonic seizures since it can worsen this seizure type [11].

In conclusion, LEV should be used with caution in children with MAE and an exacerbation of seizure frequency temporally related to the introduction of LEV should alert the clinician to the possibility of a paradoxical seizure exacerbation.

### Consent

Written informed consent was obtained from the children’s parents for publication of this Case report. A copy of the written consent is available for review by the Editor of this journal.
